# Non-thermal plasma-treated solution demonstrates antitumor activity against pancreatic cancer cells *in vitro* and *in vivo*

**DOI:** 10.1038/s41598-017-08560-3

**Published:** 2017-08-16

**Authors:** Kim Rouven Liedtke, Sander Bekeschus, André Kaeding, Christine Hackbarth, Jens-Peter Kuehn, Claus-Dieter Heidecke, Wolfram von Bernstorff, Thomas von Woedtke, Lars Ivo Partecke

**Affiliations:** 1 Department of General, Visceral, Thoracic and Vascular Surgery, University Medicine Greifswald, Sauerbruchstrasse, 17475 Greifswald, Germany; 2 Leibniz-Institute for Plasma Science and Technology (INP Greifswald), ZIK plasmatis, Felix-Hausdorff-Strasse 2, 17489 Greifswald, Germany; 3 Department of Experimental Radiology, University Medicine Greifswald, Sauerbruchstrasse, 17475 Greifswald, Germany; 4 Department of Hygiene and Environmental Medicine, University Medicine Greifswald, 17475 Greifswald, Germany

## Abstract

Pancreatic cancer is associated with a high mortality rate. In advanced stage, patients often experience peritoneal carcinomatosis. Using a syngeneic murine pancreatic cancer cell tumor model, the effect of non-thermal plasma (NTP) on peritoneal metastatic lesions was studied. NTP generates reactive species of several kinds which have been proven to be of relevance in cancer. *In vitro*, exposure to both plasma and plasma-treated solution significantly decreased cell viability and proliferation of 6606PDA cancer cells, whereas mouse fibroblasts were less affected. Repeated intraperitoneal treatment of NTP-conditioned medium decreased tumor growth *in vivo* as determined by magnetic resonance imaging, leading to reduced tumor mass and improved median survival (61 vs 52 days; *p* < 0.024). Tumor nodes treated by NTP-conditioned medium demonstrated large areas of apoptosis with strongly inhibited cell proliferation. Contemporaneously, no systemic effects were found. Apoptosis was neither present in the liver nor in the gut. Also, the concentration of different cytokines in splenocytes or blood plasma as well as the distribution of various hematological parameters remained unchanged following treatment with NTP-conditioned medium. These results suggest an anticancer role of NTP-treated solutions with little to no systemic side effects being present, making NTP-treated solutions a potential complementary therapeutic option for advanced tumors.

## Introduction

Annually, pancreatic cancer causes over 330.000 deaths worldwide^1^. Pancreatic ductal adenocarcinoma (PDA) is the most common entity of pancreatic malignancies, showing a very poor prognosis with a 5-year survival rate of about 6%^2^. Due to its aggressive growth, pancreatic cancer frequently infiltrates surrounding tissue, escaping curative surgical resections. Today’s standard chemotherapy involves systemic injections of either gemcitabine containing combinations, or FOLFIRINOX, however, leading to still unsatisfactorily prolonged survival^3–5^. Moreover, the incidence as well as annual deaths of pancreatic cancer are rising^6^, substantiating the need for new therapeutic options.

Non-thermal plasma is an ionized gas that can be applied to cells and tissues avoiding thermal damage^7^. Previously, it has been shown to inactivate various types of tumor cells *in vitro*, such as glioblastomas^8^, lung cancer^9^, leukemic cells^10^, colon cancer^11^, melanomas^12^, and pancreatic cancer^13^. The redox homeostasis in cancer is often disturbed^14^, providing a therapeutic window for NTP-mediated oxidative damage^15^. Yet, most *in vivo* experiments have been carried out using immune-compromised mice or heterotopic respectively allogeneic tumor implantation, restricted final conclusions to clinical settings. Moreover, a limited number of studies have focused on investigating the role of NTP in pancreatic cancer treatment, especially considering its complex pathogenesis and metastatic lesions in the peritoneal cavity.

Recently, NTP-treated solutions have been suggested as an alternative to direct plasma in cancer treatment^16, 17^. Due to its stability and crucial role in multiple cellular pathways^18–20^, H_2_O_2_ is assumed to be the main factor in preserving and mediating the cytotoxicity of NTP^21, 22^. However, synergistic effects with O_2_^−^ or NO_2_^− ^^23, 24^ and even H_2_O_2_ independent apoptosis have been observed^25^. All in all, NTP’s chemistry in solutions is poorly understood, yet^26^. This lack of knowledge notwithstanding, the anti-cancer capacity of NTP-treated solutions has been investigated in several studies. Both, caspase-dependent and caspase-independent apoptosis could have been induced in malignant cells^17, 27^. In an *in vivo* setting, the formation of intraperitoneal metastasis could have been reduced by intraperitoneal administration of NTP-treated medium^28^, making NTP-treated solutions a promising treatment option for disseminated cancers.

In this study, the efficacy and safety of the application of an atmospheric pressure argon plasma jet against pancreatic cancer *in vitro* and *in vivo* was explored. Its plasma generation and species output in the gas phase are well characterized^29^. Due to large number and small size of metastasis in the clinical setting, the direct NTP-treatment of numerous metastases *in vivo* seems to be impractical. Therefore, we investigated the antitumor action of NTP-treated cell culture medium that was repeatedly applied to mice challenged with 6606PDA tumor cells, being in contact with both tumor nodes but also physiological tissues. Innovative therapeutic approaches, such as this oxidative lavage, need to be tolerated well by the organism, and its benefits should outweigh the risks. With this in mind, we have investigated the efficacy of NTP-treated medium on cancer lesions and possible side effects in healthy tissues and whole blood. This study provides promising results and underlines an encouraging role of NTP-conditioned solutions in the multimodal treatment of malignancies in future therapies.

## Results

### Non-thermal plasma decreased metabolic activity and proliferation *in vitro*

**Figure 1:**
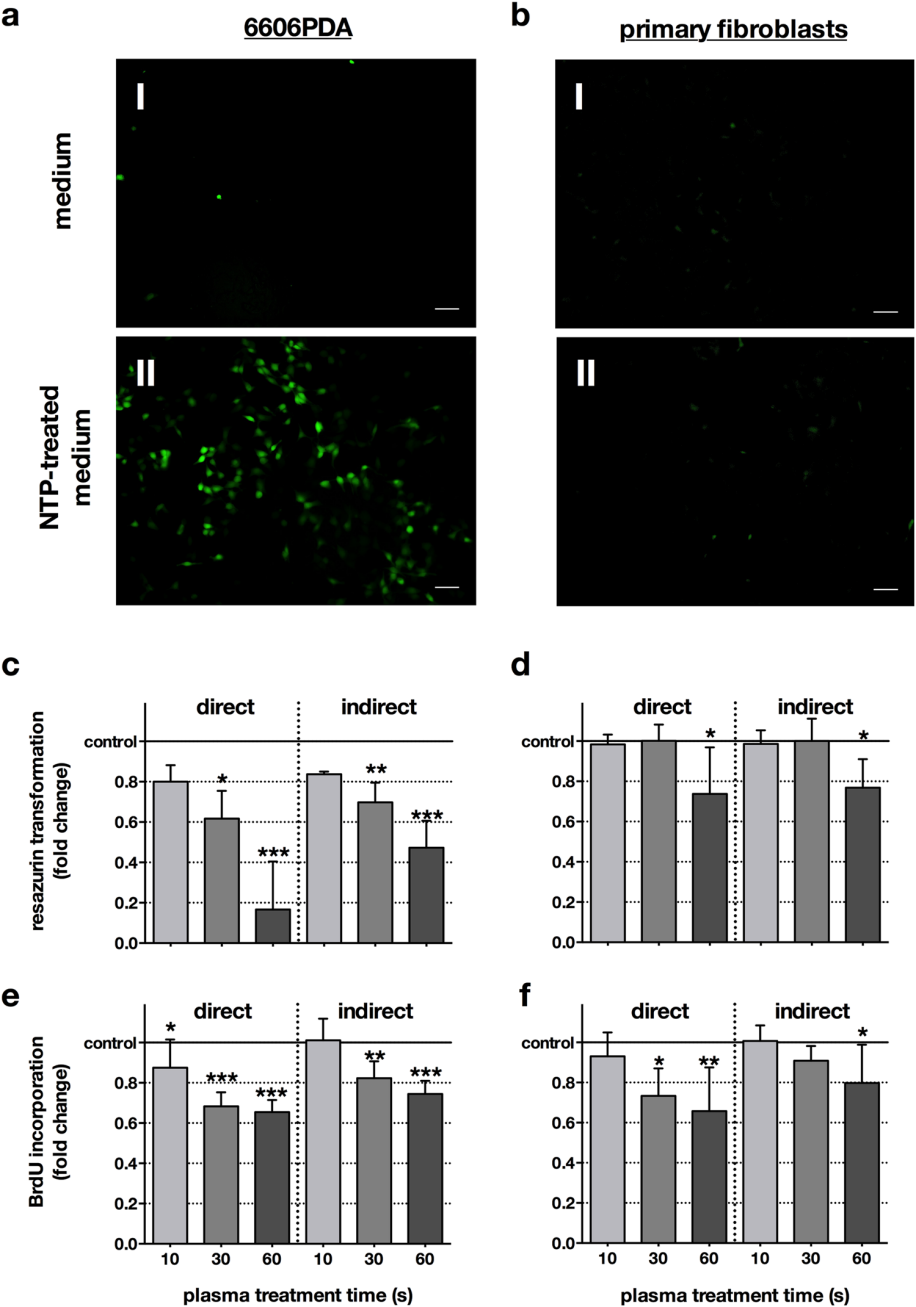
Directly applied NTP as well as NTP-treated medium conferred oxidative stress and reduced metabolic activity and cell proliferation. 6606PDA cells (**a**) and fibroblasts (**b**) were stained with CM-H_2_DCF-DA and subsequently exposed to argon gas-treated (control, I) or NTP-treated (II) medium (60 s). The former had no effect on the redox status of the cells whereas the latter strongly oxidized 6606PDA tumor cells (**a** II) but not fibroblasts (**b** II). Representative images of two independent experiments are shown. Scale bars represent 50 µm. 6606PDA cells (**c**) or fibroblasts (**d**) were directly exposed to NTP (direct) or to NTP-treated medium (indirect) and their metabolic activity was assessed after 24 h. To examine cellular proliferation, 6606PDA cells (**e**) or fibroblasts (**f**) were exposed to NTP or NTP-treated medium and cellular BrdU incorporation was quantitatively measured 4 h later. Data are presented as mean + S.D. of three to five independent experiments.

**Figure 2:**
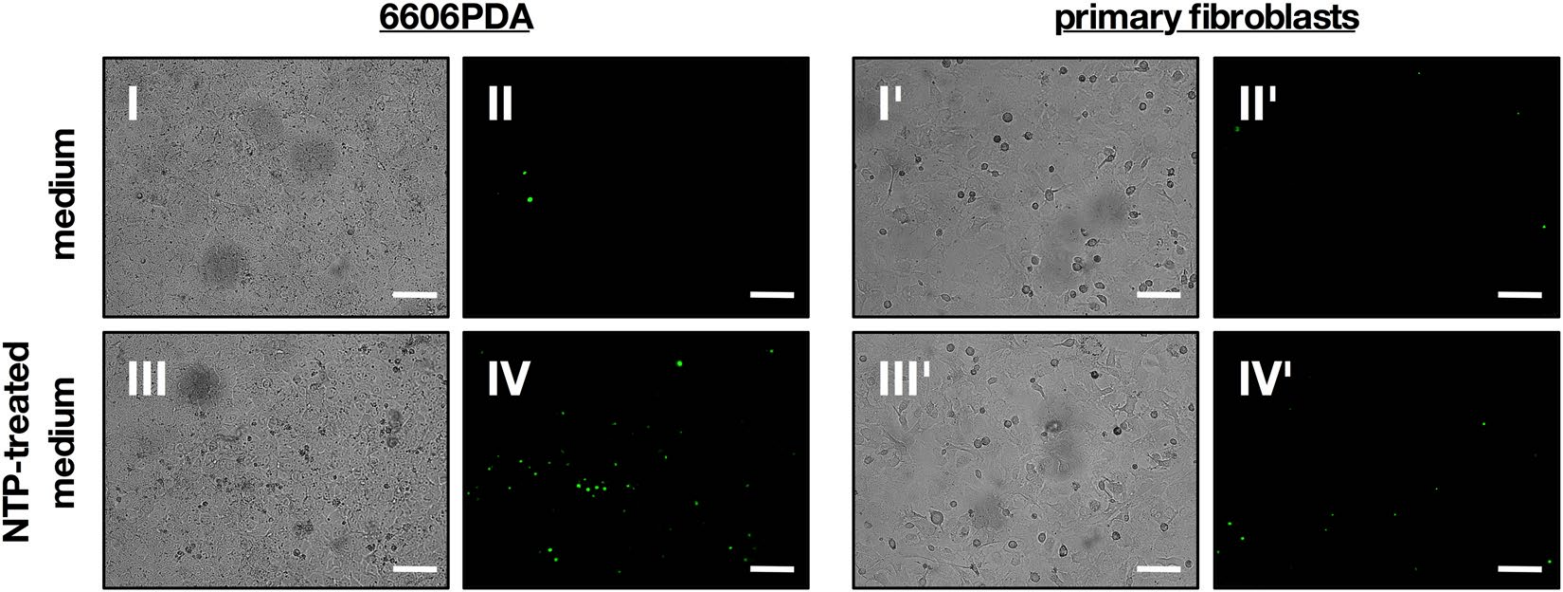
Directly applied NTP as well as NTP-treated medium induced apoptosis more effectively in 6606PDA. 6606PDA cells (left panel) or primary, murine fibroblasts (right panel) were exposed to argon gas-treated medium (control) or NTP-treated medium. After an incubation of 24 h, cells were imaged for activated caspases 3/7 by fluorescence microscopy. Representative images of two independent experiments are shown. Scale bars represent 100 µm.

NTP expels reactive species which are known to confer oxidative stress. Oxidative action was shown by a strong increase of DCF fluorescence in the 6606PDA cells exposed to NTP-treated medium but not in the control samples (Fig. 1a) and not in fibroblasts either, even after addition of NTP-treated solution (Fig. 1b). Next, the different effects on 6606PDA cells and autologous fibroblasts were examined. Therefore, we distinguished between cell viability on the one hand, detected by metabolic activity, and cell proliferation, detected by direct inhibition of DNA synthesis, on the other hand. The cells’ metabolic activity, determined by Resazurin-degradation, decreased in a treatment time-dependent fashion in both cell types but 6606PDA cells showed a significantly stronger reduction following direct or indirect NTP-application compared to fibroblasts (10 s *p* < 0.001, 30 s *p* < 0.001, 60 s *p* < 0.0028; Fig. 1c,d). Cell proliferation, identified by BrdU-incorporation, was investigated in either cell type and was significantly reduced in both treatment regimens for 6606PDA cells (direct: 10 s *p* < 0.048, 30 s *p* < 0.001, 60 s *p* < 0.001; indirect: 30 s *p* < 0.003, 60 s *p* < 0.001; Fig. 1e) and fibroblasts (direct: 30 s *p* < 0.024, 60 s *p* < 0.004; indirect: 60 s *p* < 0.030; Fig. 1f), alike. Differences between the direct and indirect treatment regimen were not significant, suggesting NTP-treated medium being suitable for treatment of pancreatic lesions *in vivo*. We then investigated the mode of cell death and found induction of apoptosis to be present in 6606PDA cells and, to a lesser extent, in fibroblasts 24 h after exposure to NTP-treated medium (Fig. 2).

### NTP-treated medium improved survival by reducing tumor burden through apoptosis

**Figure 3:**
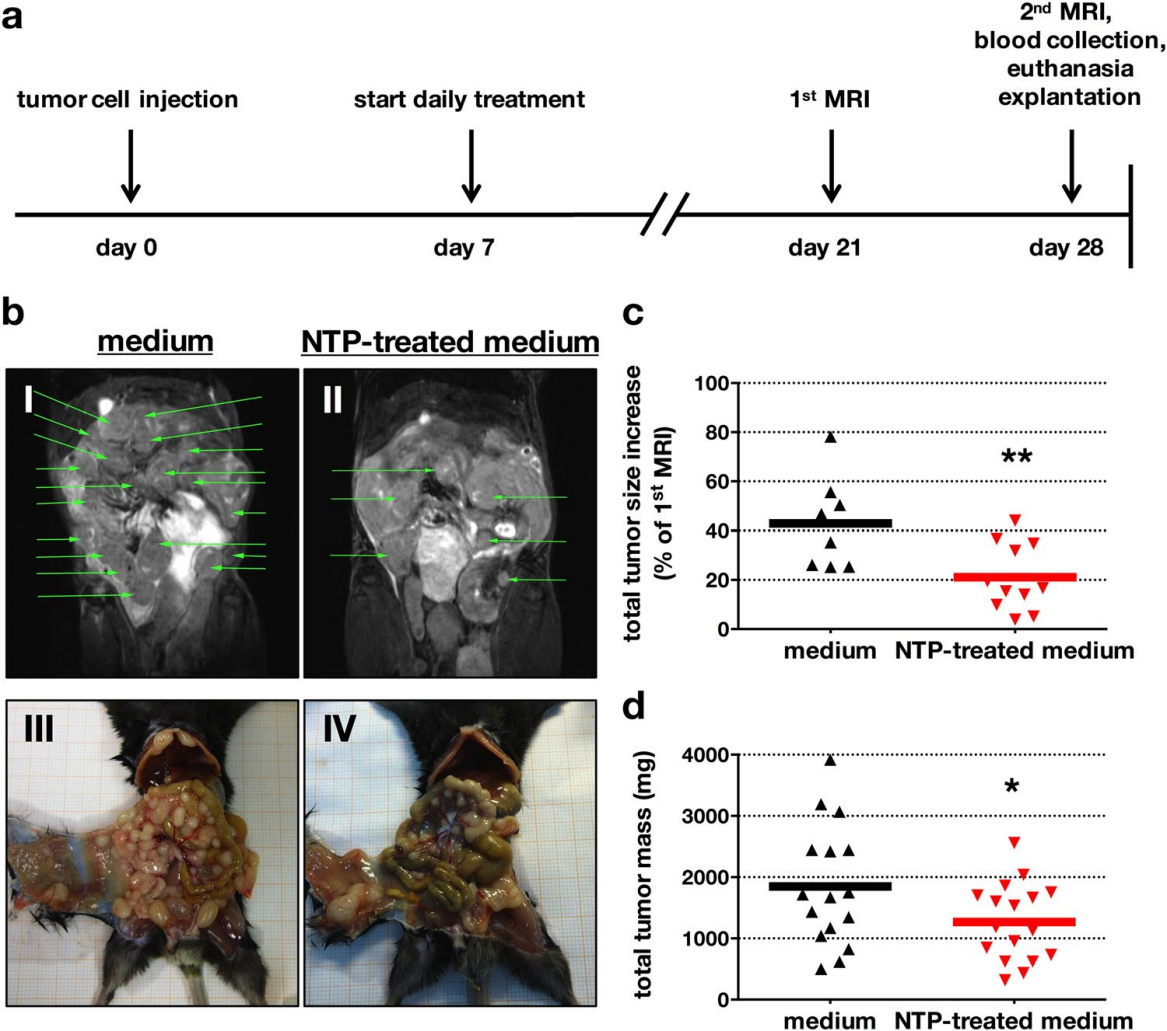
NTP-treated medium decreased number, growth, and size of pancreatic lesions *in vivo*. The experimental timeline (**a**) is given. The number of tumor nodes in the peritoneum was followed by MRI and was elevated in the control animals (I) compared to NTP-treatment group (II) at the end of treatment (d28). Also, corresponding macroscopic findings for control (III) and NTP-treated (IV) animals are shown (**b**). MRI-based calculation of tumor volume revealed a significantly decreased total tumor growth in the treatment group (**c**). At day 28, animals were sacrificed and tumor nodes were excised and weighed, showing a significantly decreased total tumor mass (**d**). Representative images of 13 animals are given, green arrows indicate tumor nodes (one green arrow per lesion) (**b**). Data are presented as mean of 8–17 animals (**c**,**d**).

**Figure 4:**
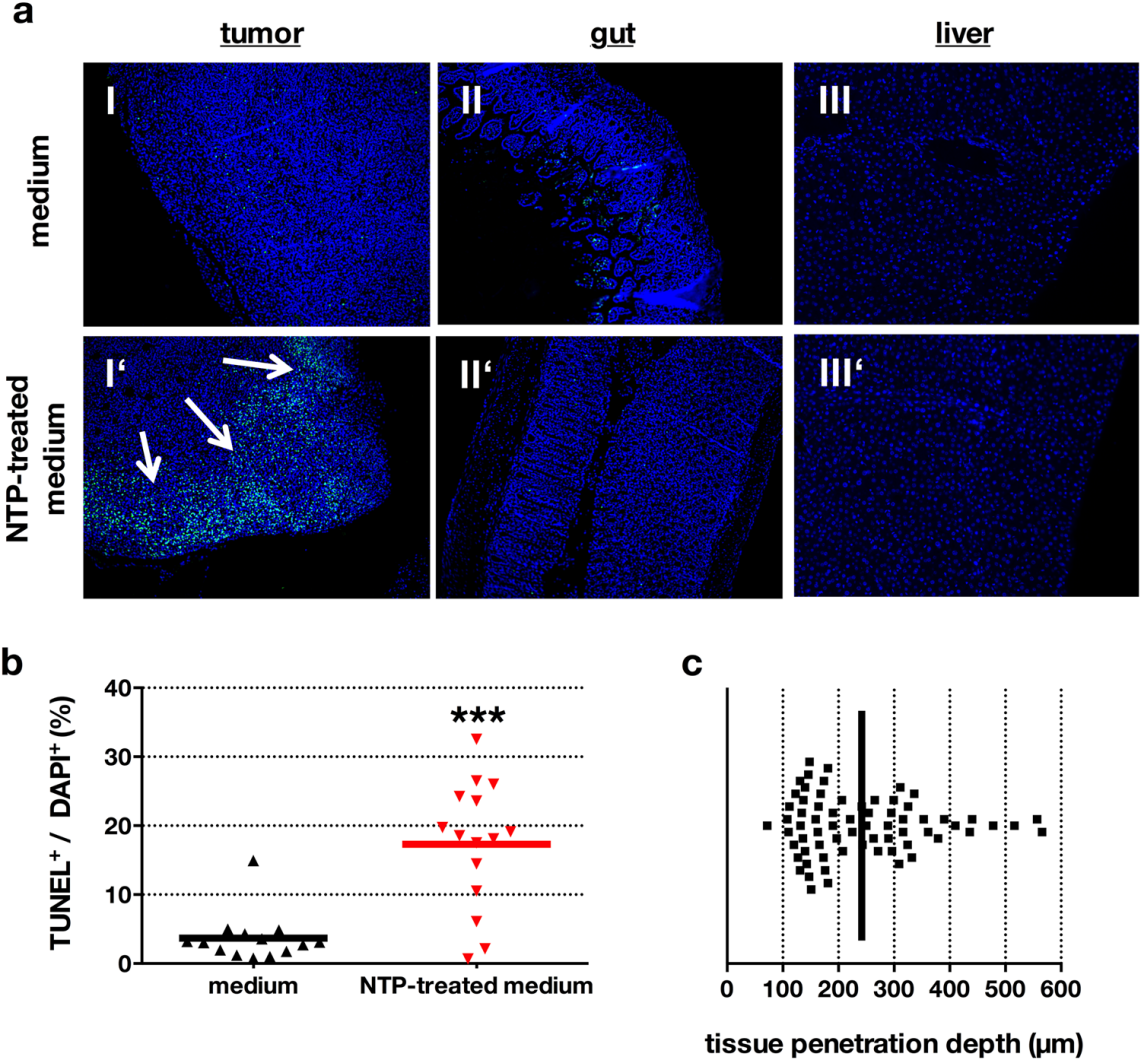
NTP-treated medium induced apoptosis in tumor tissue but not in the gut or liver. Tumor-bearing mice were sacrificed and tumor nodes (I, I′), gut (II, II′) and liver (III, III’) were removed. Paraffin tissue-sections were made and stained with TUNEL for apoptotic cells and DAPI for nuclei (**a**). TUNEL^+^/DAPI^+^-quotient was significantly elevated in mice receiving NTP-treated medium (**b**). The depth of apoptotic cells in the tissue margin of several sections and animals was quantified (**c**). Black line indicates mean value of tissue penetration. Tissue samples are presented as one representative of fifteen different animals (**a**). Data are presented as mean of several values per section (**b**,**c**).

**Figure 5:**
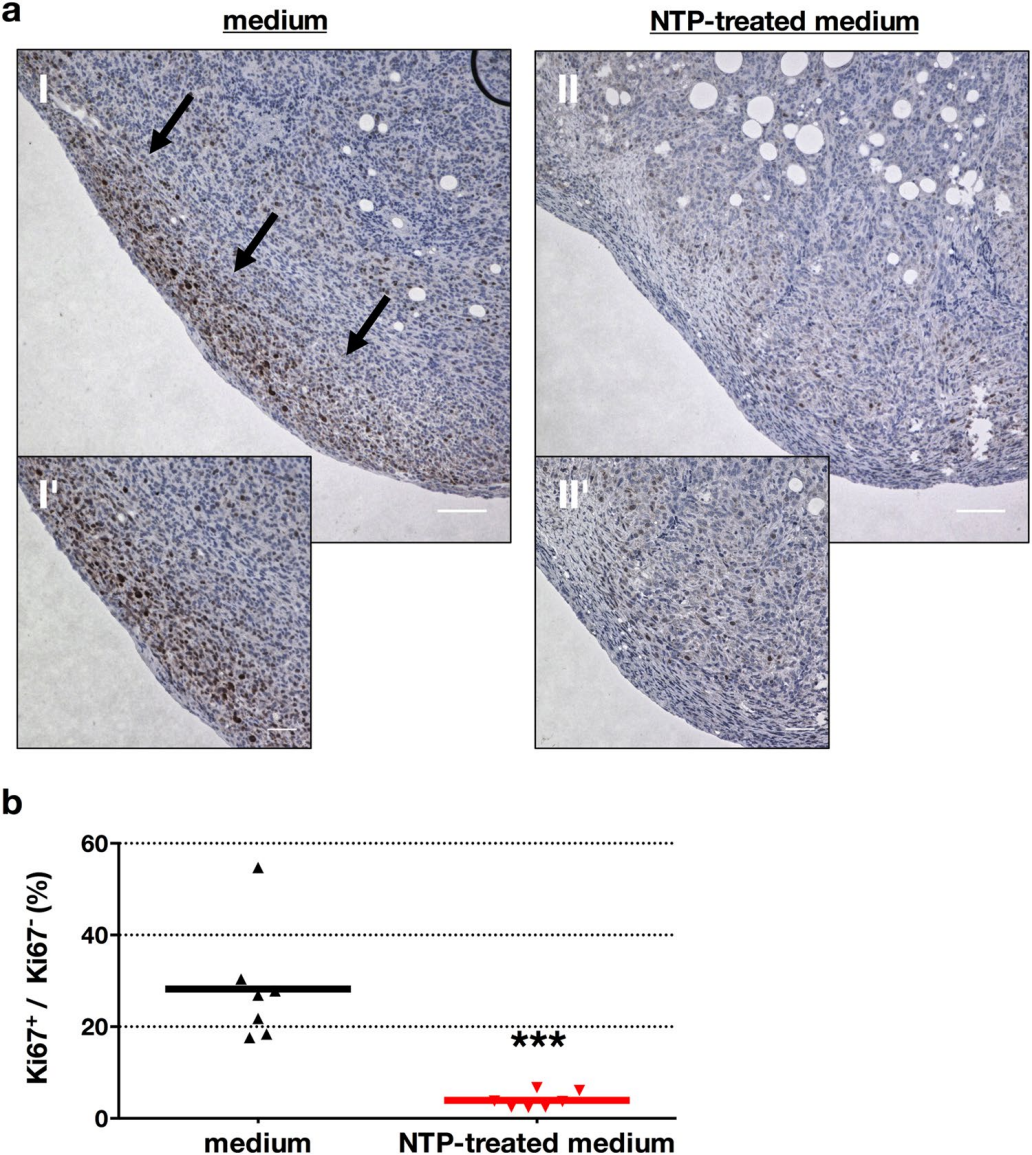
NTP-treated medium abrogated cancer cell proliferation *in vivo*. Tumor-bearing mice were sacrificed after receiving untreated or NTP-treated medium for 21 consecutive days. Tissue sections of tumors were stained for the proliferation marker Ki67. Microscopy revealed proliferation at the tumor margin in the control (**a** I, I′, arrows) but not the treatment group (II, II′) and the difference was significant (**b**). Representative images (**a**) or mean of at least 5 different tumor nodes per mouse (**b**) are shown. Scale bars represent 100 µm (I, II) and 50 µm (I’, II’).

To address antitumor effects of NTP-treated medium, (Fig. 3a) MRI monitoring was employed to quantify tumor growth (Fig. 3b). After sacrificing animals, tissue sections were performed. Tumor growth was significantly (*p* < 0.008) decreased in the treatment group by 21.1 ± 4.1% (Fig. 3c), and tumor resections showed a significantly decreased (*p* < 0.029) total tumor weight by 31.3% in the treatment group (Fig. 3d). Both findings pointed to a therapeutic effect of NTP-conditioned medium. We next asked how this effect was mediated with NTP-treated medium. TUNEL staining of tumor nodes (Fig. 4a) revealed significant (*p* < 0.001) apoptotic responses being present in tumor margins of mice receiving NTP but not control medium (Fig. 4b). Interestingly, extensive apoptosis was also present in deeper layers of the tumor tissue (mean = 242 µm), suggesting a considerable deep penetration of the NTP-treated medium into the malignant tissue (Fig. 4c). By contrast, no apoptosis was found in tissue sections of the gut and liver (Fig. 4a), suggesting the NTP-treatment to have selective effects against tumors. At the same time, tumor margins of the control group showed strong cell proliferation, whereas tumor margins in the NTP group did not (Fig. 5a). Quantitative evaluation (Fig. 5b) revealed a significant difference in total proliferation (*p* < 0.001). These results suggested an efficacy of NTP-conditioned medium in tumor reduction by inducing apoptosis and reducing proliferation.

**Figure 6:**
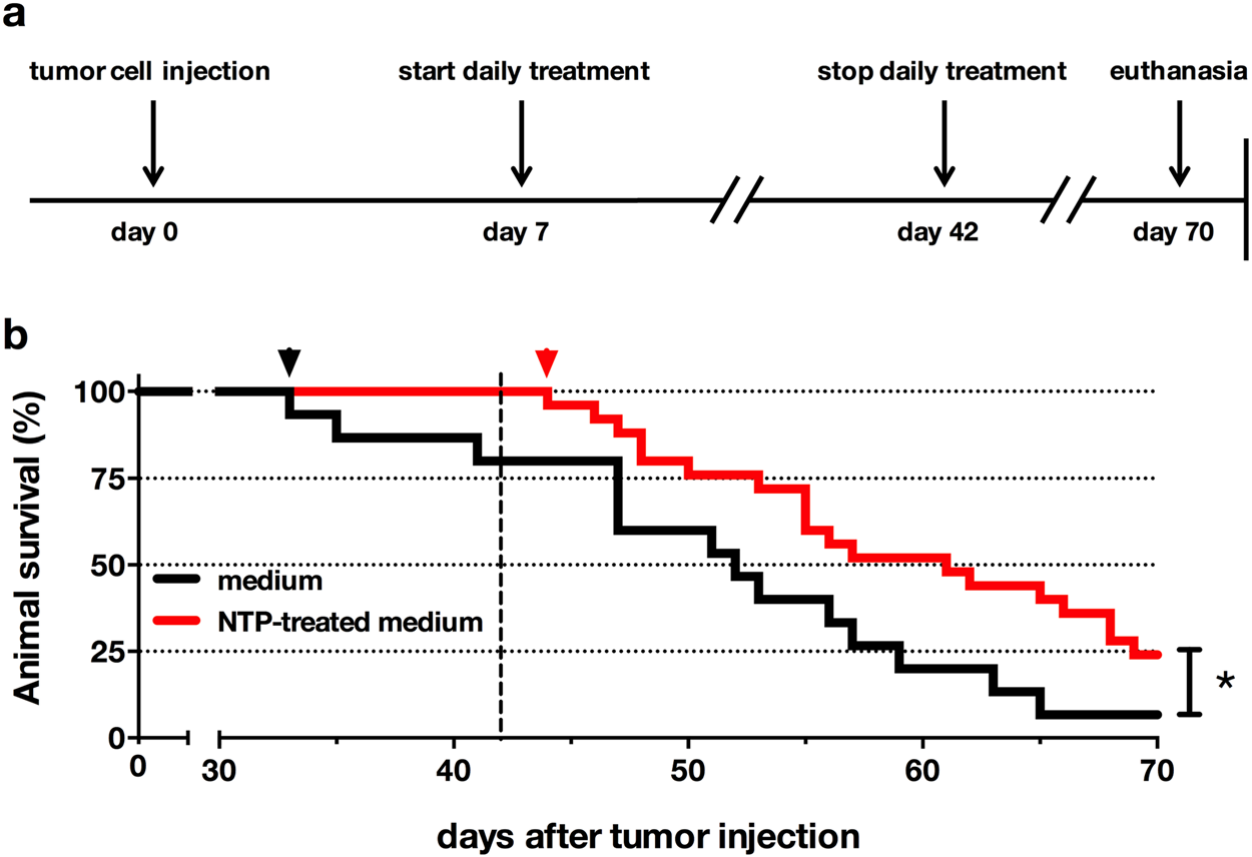
NTP-treated medium significantly enhanced survival in tumor-bearing animals. The experimental timeline for animal survival is given (**a**). Lethal tumor growth (**b**) showed a later onset (arrowhead) in the treatment (44 days) compared to the control group (33 days). Overall survival was significantly enhanced. Kaplan-Mayer survival curve is representative of 25 animals (NTP-treated medium) or 15 animals (medium), respectively. Dashed line indicates last treatment day.

To address potential beneficial effect of NTP-treated medium to animal survival, we monitored the viability of tumor-bearing control and NTP treated mice (Fig. 6a). Above all, median survival was increased in the NTP group (61 days) compared to untreated control animals (52 days, *p* < 0.024; Fig. 6b). Moreover, not one of the NTP receiving animals died during therapy, leading to much later onset of animal death in the treatment group (44 days) compared to control animals (33 days).

### Systemic side effects of NTP-treated medium were not present *in vivo*

**Figure 7:**
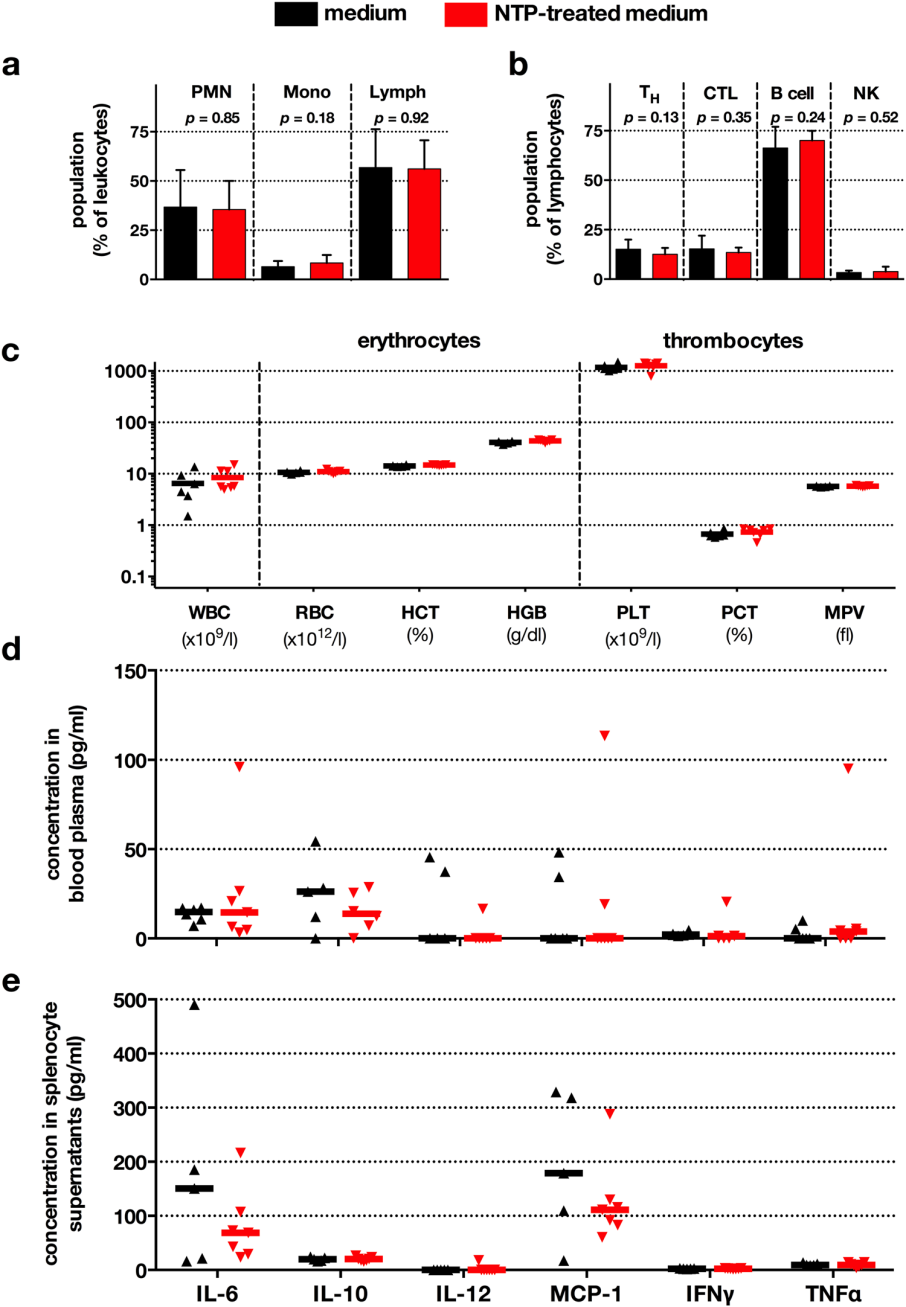
Blood parameters, leukocyte distribution, and cytokine signatures remained largely unaffected. Tumor-bearing mice were sacrificed after receiving untreated or NTP-treated medium for 21 consecutive days. Blood leukocyte distribution was determined by multicolor flow cytometry (**a**,**b**). Total leukocyte counts and erythrocyte and thrombocyte parameters were obtained by VetScan measurement of whole blood (**c**). Data are presented as mean + S.D. of 13 animals (**a**,**b**) or 6-7 animals (**C**). PMN = polymorph-nuclear cells; Mono = monocytes; Lymph = lymphocytes; T_H_ = T helper cells; CTL = cytotoxic T cells; NK = natural killer cells; WBC = white blood cells; RBC = red blood cells; HGB = hemoglobin; HCT = hematocrit; PLT = platelets; PCT = platelet hematocrit; MPV = mean platelet value. Cytokine concentrations were determined in blood plasma using cytometric bead array and flow cytometry (**d**). Splenocytes from both treatment groups were obtained from mice sacrificed at d28 (**e**). Cytokine concentrations in cell culture supernatants were assessed as for blood plasma. Data are presented as median of 6–7 animals per group.

NTP-treated medium was active against tumors *in vitro* and *in vivo*. Another prerequisite for an envisioned therapeutic application of NTP-treated solutions is its safe use. We therefore studied whether systemic side effects occurred after 21 consecutive injections of NTP-conditioned medium. Daily visual inspection of mice did not reveal an additional ill-being or altered behavior of mice receiving NTP-treated medium. Blood was collected to assess systemic side effects by studying the blood leucocyte content indicative of systemic changes in inflammation or stress. Using ten-color flow cytometry of stained and lysed whole blood, we did neither find any significant differences in the percentages of granulocytes, monocytes, and lymphocytes in general (Fig. 7a), nor of T helper cells, cytotoxic T cells, B cells, and NK cells in particular (Fig. 7b). Multiparametric analysis of mouse whole blood confirmed and extended this finding to number, size, shape, volume, and distribution of erythrocytes as well as thrombocytes (Fig. 7c). Inflammation is not only reflected in blood counts but also in its cytokine signature. No significant changes in the concentration of IL6, IL10, IL12, MCP1, IFNγ, or TNFα were observed (Fig. 7d). The spleen is also central in regulating inflammation and eliciting immune responses. We therefore cultured splenocytes of sacrificed mice that had received untreated or NTP-treated medium, and investigated their steady-state cytokine production. Concentrations of IL6, IL10, IL12, MCP1, IFNγ, and TNFα did not differ significantly between both groups (Fig. 7e). Taken together, these results as well as the lack of apoptotic cells in the gut or the liver (Fig. 4a) suggest a systemically non-toxic application of NTP-treated medium into the peritoneum of tumor-bearing mice.

## Discussion

Pancreatic cancer is highly aggressive and malignant. Particularly in case metastases spread into the peritoneum, therapeutic options, also in palliative settings, are limited. Chemotherapy using gemcitabine has been shown to be inappropriate in improving patients’ life quality^30^. Recently, exposure of tumor cells to non-thermal plasma has been identified to be an innovative antitumor approach^31, 32^. We here demonstrated *in vitro* a comparable cytotoxicity of both the direct and the indirect NTP-treatment. The atmospheric pressure argon plasma jet generates reactive components of many kinds, among them reactive oxygen and nitrogen species^33^. At sufficient concentrations, the latter are known to mediate cytotoxic effects, decrease tumor size, and prolong survival in mice challenged with pancreatic cancer^34^. Underscoring the dual role of ROS/RNS in health and disease^35^, however, depriving pancreatic cancer cells of endogenous ROS *in vitro* was shown to induce apoptosis as well^36^. In cell culture medium, especially long-living oxidants, such as hydrogen peroxide, and modified protein residues were discussed to be largely responsible in facilitating NTP-mediated cell damage^22, 37^. Redox-modulating strategies are of particular importance in selective anti-tumor treatment regimens^38^. Hence, it seems plausible to use NTP-treated solutions as an antitumor agent in peritoneal pancreatic adenocarcinomatosis.

A promising tumor therapy should not only be effective but also targeted. NTP-treatment was more cytotoxic to malignant cells compared to non-malignant fibroblasts. Concerning the reduction of cell proliferation, tumor cells also were more sensitive to NTP-treatment than healthy fibroblasts. This may be explained by a higher baseline ROS concentration in tumor cells, especially in k-ras mutated tumor cells^39^, which is suggested to decrease the amount of ROS/RNS needed to induce apoptosis^38, 40^. Additionally, it has been shown that the cellular H_2_O_2_ uptake depends on transmembrane aquaporins^41^ and cancer cells are known to express those channel proteins in close frequency^42^. It has been suggested, that this point is crucial in NTP-selectivity^43^. Apropos, a significantly increased rate of apoptotic cells has also been found in other cancer cell types after NTP-treatment^44–46^. However, using cultured cell lines as a model of non-malignancy *in vitro* is of limited significance as unlimited growth is *per se* not physiological and adaptions to cell culture conditions occur after numerous passages^47^. By contrast, we here used autologous fibroblasts from mice cultured for only a few passages, and compared them with pancreatic cancer cells derived from the same mouse strain, providing a good model. We found a targeted induction of apoptosis in malignant but not in non-malignant cells, substantiating the need for a selective cytotoxicity of NTP that was previously requested for NTP-based cancer treatment^32^. Additionally, we previously demonstrated a synergistic effect of NTP-treatment combined with the standard antitumor therapeutic gemcitabine *in vitro* ^48^, implementing NTP as a possible additional treatment option in multimodal anti-cancer therapy in the future.

NTP-treated medium enhanced median survival and reduced tumor burden in mice. These results are in line with a study of Vandamme and colleagues who demonstrated in an artificial *in vivo* model a 60% decrease of glioma size, hampered tumor growth, and a survival advantage in mice receiving direct NTP-treatment^49^. An *in vitro* and *in vivo* reduction of cancer growth has also recently been observed for chemo-resistant ovarian cancer^17^ and even pancreatic cancer^50^. Yet, both studies did not conclude a significant survival advantage and treatment with NTP-treated medium was carried out already 24 h after tumor cell injection rather suggesting a growth inhibition of initial tumor lesions than growth control of fully developed tumor nodes. In the present *in vivo* model, pro-apoptotic effects after exposure to NTP-treated medium were present up to 566 µm into the tumor node. This was somewhat unexpected as the direct exposure to NTP generated by the same argon plasma jet was previously shown to induce apoptosis in pancreatic cancer lesions in a Tum-CAM *in vivo* model in a depth of only up to 50 µm^13^. It is unlikely that reactive components dissolved in NTP-treated medium diffused deeply into the tumor tissue without reacting with cellular components and/or being scavenged by host antioxidants, thereby losing their activity. It has been described that exposure to NTP downregulates the expression of cell adhesion molecules^51^, effectively detaching adherent cells *in vitro* ^52^ facilitating loosening of cell-cell contacts. If such an effect was rapid, the ROS/RNS of NTP-treated medium could penetrate laterally to the less-adherent cells more deeply into the tumor tissue. Tumor cells have also previously been shown to display a different architecture of adhesion molecules^53^ that may be more prone to oxidation compared to healthy tissues. In addition, cells in solid metastases are *per se* less adherent which is reflected by their reduced formation of tight junctions^54^.

In a clinical context, a prerequisite of an ideal anti-cancer therapy would be its safe use as well as showing localized but not systemic effects. Importantly, plasma-treated solution was previously shown to be non-mutagenic *in vitro* ^55^, and significant effects on blood plasma antioxidant status could not be observed^56^. The components of the blood reflect systemic side effects. Drugs, oxidative stress, or infections induce changes in erythrocyte and thrombocyte properties^57–59^. However, we did not observe any effects in blood of NTP-treated mice. Similarly, NTP-conditioned medium did not modulate cytokine concentrations present in the blood or released by splenocytes *ex vivo*. Substantiating these results, NTP-related damage was not found in the liver or gut in treated mice, suggesting a higher sensitivity of tumor tissues than non-malignant tissues to NTP-related effects, as demonstrated *in vitro*.

The clinical significance of this study is limited in one important point: cell culture medium represents no realistic option for NTP-lavage in human patients. Yet, we do not clearly know how NTP-related effects were mediated by solutions^26^. However, further studies should focus on the suitability of clinically established solutions, just like ringer-lactate or PBS, as their anti-cancer efficacy has very recently been demonstrated^60, 61^.

Altogether, we here used a murine pancreatic cancer model employing peritoneal metastases, and observed reduced tumor burden and prolonged survival in mice after NTP-treatment. Moreover, we could not identify systemic stress responses or an altered hematopoiesis after recurrent systemic exposure to non-thermal plasma. We conclude that the application of NTP-conditioned medium may be an effective and safe treatment option for advanced gastrointestinal cancer and peritoneal metastases in the future.

## Materials and Methods

### Cell culture and syngeneic tumor animal model

The murine pancreatic cancer cell line 6606PDA (kindly gifted by Tuveson) was isolated from a pancreatic adenocarcinoma of transgenic C57BL/6 mice, carrying a KrasD12G allele^62^. Cells were subcultured twice a week in DMEM containing 10% fetal bovine serum, 100 U/ml penicillin, and 100 µg/ml streptomycin (all Gibco, USA). Primary, non-immortalized fibroblasts were obtained from C57BL/6 mice (Charles River, Germany) as described elsewhere^63^, and cultured *in vitro* under identical conditions as for 6606PDA for up to ten passages. Tumor growth in male, nine weeks old mice (average weight 24 ± 2.4 g) was elicited by intraperitoneal injection (27 G) of 2 × 10^6^ 6606PDA cells dissolved in 1 ml of medium into the lower left abdominal quadrant. The tumors were allowed to grow for seven days prior to therapeutic interventions. This setting resulted in the development of manifest and diffuse peritoneal carcinomatosis in all four abdominal quadrants. Animal health and weight was monitored daily.

### NTP-treatment *in vitro* and *in vivo*

NTP was generated by the atmospheric pressure argon plasma jet *kINPen MED* (neoplas tools GmbH, Germany). Within its chassis, a rod-like electrode is mounted which is shielded by a dielectric capillary. Using three standard liters of argon flow (Air Liquide, France), the NTP was ignited by generating a sinusoidal voltage with a frequency of about 1 MHz^29, 64^. For NTP-treatment *in vitro*, cells in 100 µl of medium were seeded in 96 well plates and the jet was hovered at a constant distance of 5 mm over the center of each well for the indicated time length (direct approach). For exposure to NTP-treated medium (indirect approach), 120 µl of DMEM were treated in 96 well plates and 100 µl were consecutively added to cells in 96 well plates of which the medium has been removed before. Samples receiving treatment with argon gas alone were always similar to untreated controls and for reasons of simplicity not graphed. For *in vivo* experiments, NTP-treated medium was generated by exposing 5 ml of DMEM in 60 mm dishes to plasma for 10 minutes. Evaporation of the liquid due to the gas flux was compensated by addition of a predetermined amount of double-distilled water. For treatment, cages (5 animals per cage) were randomly grouped to a treatment regimen or controls. One group received 1 ml of untreated DMEM while the other one received NTP-treated DMEM (1 ml) via intraperitoneal injection on a daily basis and for up to 35 days.

### Oxidation, metabolic activity, cell proliferation, and apoptosis measurement in cell cultures

To assess oxidation in 6606PDA, cells were loaded with 5 µM CM-H_2_DCF-DA (life technologies, USA) for 20 min in the incubator and washed prior to exposure to NTP-conditioned medium. Subsequently, fluorescence images were acquired using an epifluorescence microscope (Zeiss, Germany). The metabolic activity of 6606PDA was assessed using resazurin (Promega, USA). Briefly, 2 × 10^3^ cells per well were seeded into 96 well plates and allowed to adhere overnight. Subsequently, cells were exposed either directly to NTP or to NTP-treated medium, and incubated for 4 h before receiving fresh medium. After another 20 h of incubation, 20 µl of resazurin was added and resorufin fluorescence (λ_ex_ 560 nm, λ_em_ 590 nm) was measured using a plate reader (Tecan, Switzerland). For evaluation of cell proliferation, 5 × 10^4^ cells per well were seeded into 96 well plates followed by either direct NTP-treatment or treatment with NTP-conditioned medium on the next day. After 2 h of incubation, cells received fresh medium containing the pyrimidine analog 5′-bromo-2′-deoxyuridine (BrdU; Cell Signaling Technology, USA) and were incubated another 2 h. After BrdU incorporation, cells were fixed and permeabilized, and proliferation was measured using a detection antibody coupled to horseradish peroxidase catalyzing a color change in TMB (absorbance λ = 450 nm) which was quantified using a plate reader. Apoptosis was assessed by exposing 6606PDA or fibroblasts to NTP-conditioned medium. Cells were then incubated for 24 h followed by the addition of a caspase 3/7 detection reagent (life technologies) and fluorescence microscopy.

### Tumor mass and *in vivo* tumor growth determination using MRI

The tumor mass was determined after 21 days of consecutive exposure to NTP-treated or untreated medium (Fig. 3a). Tumor growth in living mice was determined by magnetic resonance imaging (MRI) using a 7.1 tesla *ClinScan* device (Bruker, Germany) as previously described in detail^65, 66^. 12 hours prior to imaging, food was removed and water mixed with sorbitol (2:1) improving abdominal image quality. At date of examination, mice were anesthetized with isoflurane (1%). Heart rates, body temperature, and respiratory rates were constantly monitored. Only intraperitoneal metastases (index lesions) were chosen that could be confidently identified at day 21 and day 28 in both the coronal as well as the axial image plane. The maximum diameter was calculated for both days and normalized to express the tumor growth in percent. Image analysis was performed using *OsiriX* software (Pixmeo SARL, Switzerland). Subsequently, animals were sacrificed, and tumor nodes of the peritoneum were excised, collected, and weighed.

### Blood parameters, leukocytes, cytokines

Prior sacrifice, mice were anesthetized with isoflurane (1%), and blood was collected via retro-orbital puncture. One-hundred microliter of blood were subjected to automated blood parameter analysis using a *VetScan* hematology device (Abaxis, USA). Also, 100 µl of blood were incubated with TruStain FX (BioLegend, USA) to block Fc-receptors, and stained with ten different monoclonal antibodies (clone) directed against surface molecules of major leukocyte subpopulations: Ly6C FITC (HK1.4), NK-1.1 PE (PK136), CD11b PE-Dazzle (M1/70), CD8a PerCPCy5.5 (536.7), CD3 PECy7 (17A2), CD43 APC (S11), CD4 AlexaFluor700 (RM45), B220 APCCy7 (RA36B2), CD115 BV421 (AFS98), Ly6G BV510 (1A8); all BioLegend. Red blood cells were lysed using an ammonium chloride-based buffer (BioLegend), and pellets washed with FACS buffer. Samples were acquired on a three-laser, ten-color *Gallios* flow cytometer (Beckman-Coulter, USA). Data analysis was performed using *Kaluza 1.5* software (Beckman-Coulter). For cytokine analyses, blood plasma was obtained by centrifugation, and stored at −80 °C until analysis using cytometric bead array (Becton-Dickinson, USA) which was acquired on a *LSRII* cytometer (Becton-Dickinson). Also, splenocytes were recovered from sacrificed mice and incubated *in vitro* overnight. Supernatants were taken off and analyzed using cytometric bead array.

### Tissue sections and immunofluorescence

After animal sacrifice by cervical dislocation, tumors and multiple organs were resected and embedded in paraffin. Four micrometer sections were generated followed by immunohistochemistry: Sections were fixed with 4% paraformaldehyde (Sigma), washed, and stained with terminal deoxynucleotidyl transferase dUTP nick end labeling (TUNEL) indicative of apoptotic cells (life technologies, USA), and 4′,6-diamidino-2-phenylindole (DAPI) for labelling cell nuclei. The depth of tissue penetration in tumor nodes was determined by detecting the deepest apoptosis cluster (n = 14 mice with five measurements per tumor node). Proliferating cells were stained with anti-mouse Ki-67 (IHC00375; Bethyl, USA) and HRP labelled anti-rabbit antibodies (K4002; Dako, USA), and DAB substrate (Dako) was added before hematoxylin staining and mounting of samples on microscopy slides using Faramount (Dako). For determination of the cell proliferation index, Ki-67 positive cells and Ki-67 negative cells were counted manually. Tissue sections were microscopically investigated using a *Keyence BZ-9000* microscope and evaluated with *BZ-II-Analyzer 4.6.2.2* software (Keyence, Japan).

### Statistics

Statistical analysis was carried out using *prism 6.07* (graph pad software, USA). To compare different treatment groups in proliferation and resazurin assays, one-way analysis of variances (ANOVA) with *Dunnett* correction to untreated controls was employed. To compare treatments between cell types, and the direct and indirect NTP-application as well as tumor growth, tumor weight, and the percentage of apoptotic or proliferating cells in tumor lesions, unpaired *t*-test was employed. Animal survival was compared using Gehan-Breslow-Wilcoxon test. *Student’s t-*test was used for comparison of leukocyte populations and parameters of whole blood analysis. Statistical differences in cytokine experiments were validated using two-way ANOVA with Holm-Sidak post testing. Significance levels were indicated as follows: *α = 0.05, **α = 0.01, and ***α = 0.001.

### Study approval

Mouse maintenance and experimental procedure were thoroughly reviewed and received approval by the State agency for agriculture, food safety, fishery Mecklenburg-Vorpommern (LALLF-MV, application number 7221.3-1.1-003/14). All animal procedures were performed in accordance with the relevant guidelines and all efforts were made to minimize suffering.

## Electronic supplementary material

Supplementary Information
