# Supplementary material for: Non-thermal plasma-treated solution demonstrates antitumor activity against pancreatic cancer cells in vitro and in vivo
Source: Sci Rep. 2017 Aug 16;7:8319. doi: 10.1038/s41598-017-08560-3 (PMC5559449; doi:10.1038/s41598-017-08560-3)
Supplement: Supplementary file 1 — Supplementary Information [file 41598_2017_8560_MOESM1_ESM.docx]

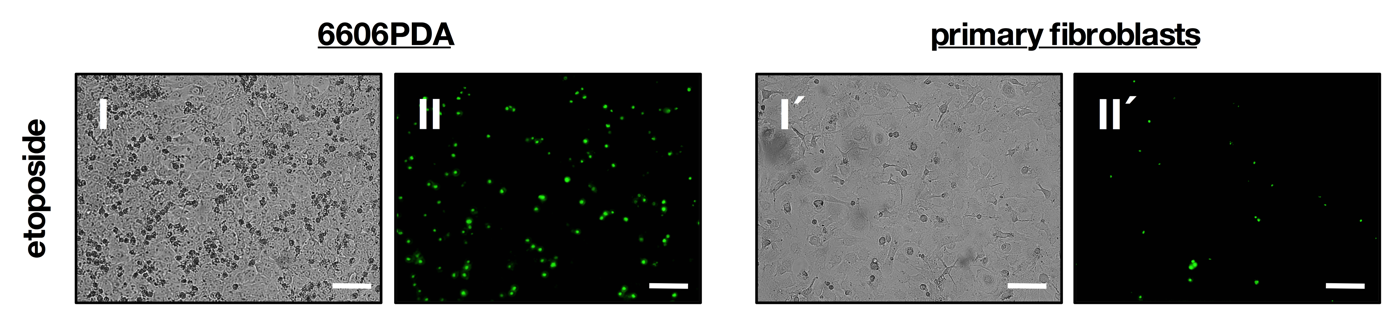


**Supplemental data (referring to Fig. 1):** Positive control for apoptosis induction via etoposide.

6606PDA cells (left panel) or primary, murine fibroblasts (right panel) were exposed to medium containing apoptosis-stimulating etoposide (50 µmol/l), additionally to argon gas-treated or NTP-treated medium (Fig. 1). After an incubation of 24 h, cells were imaged for activated caspases 3/7 by fluorescence microscopy. Representative images of two independent experiments are shown. Scale bars represent 100 µm.
